# Particulate Matter, an Intrauterine Toxin Affecting Foetal Development and Beyond

**DOI:** 10.3390/antiox10050732

**Published:** 2021-05-06

**Authors:** Hui Chen, Brian G. Oliver, Anushriya Pant, Annabel Olivera, Philip Poronnik, Carol A. Pollock, Sonia Saad

**Affiliations:** 1 School of Life Sciences, Faculty of Science, University of Technology Sydney, Sydney, NSW 2007, Australia; brian.oliver@uts.edu.au (B.G.O.); Annabel.Oliveira@student.uts.edu.au (A.O.); 2 School of Medical Sciences, Faculty of Medicine and Health, University of Sydney, Sydney, NSW 2006, Australia; apan6079@uni.sydney.edu.au (A.P.); philip.poronnik@sydney.edu.au (P.P.); 3 Renal Research Laboratory, Kolling Institute of Medical Research, Sydney, NSW 2065, Australia; carol.pollock@sydney.edu.au

**Keywords:** PM, foetal programming, in utero, neurological, respiratory, renal, endocrine, preventative treatment

## Abstract

Air pollution is the 9th cause of the overall disease burden globally. The solid component in the polluted air, particulate matters (PMs) with a diameter of 2.5 μm or smaller (PM_2.5_) possess a significant health risk to several organ systems. PM_2.5_ has also been shown to cross the blood–placental barrier and circulate in foetal blood. Therefore, it is considered an intrauterine environmental toxin. Exposure to PM_2.5_ during the perinatal period, when the foetus is particularly susceptible to developmental defects, has been shown to reduce birth weight and cause preterm birth, with an increase in adult disease susceptibility in the offspring. However, few studies have thoroughly studied the health outcome of foetuses due to intrauterine exposure and the underlying mechanisms. This perspective summarises currently available evidence, which suggests that intrauterine exposure to PM_2.5_ promotes oxidative stress and inflammation in a similar manner as occurs in response to direct PM exposure. Oxidative stress and inflammation are likely to be the common mechanisms underlying the dysfunction of multiple systems, offering potential targets for preventative strategies in pregnant mothers for an optimal foetal outcome.

## 1. Particulate Matter (PM)—An Intrauterine Toxin

Embryonic and foetal development is sensitive to the in utero environment, e.g., maternal stress, poor nutrition and environmental toxins [1,2,3,4]. A poor intrauterine environment is notably correlated with low birth weight in the offspring. The Barker hypothesis links events in foetal development, such as intrauterine growth restriction, to the increased susceptibility to develop future adult diseases [5,6,7]. In recent years, the importance of intrauterine environmental factors has been increasingly recognised in the postnatal susceptibility to non-communicable illnesses, including respiratory disorders, metabolic disorders, cardiovascular diseases and chronic kidney disease [1,8].

Apart from the abovementioned well-accepted factors causing foetal underdevelopment, air pollution has also been increasingly recognised as a major intrauterine toxin [9,10]. The World Health Organisation (WHO) has raised the alarms regarding the gravity of poor air quality on human health in the global setting, based on the studies suggesting the detrimental health effects of direct exposure to PMs derived from fossil fuel, biomass burning and traffic [11]. The burden of PM on health is unevenly distributed. Pregnant women and their unborn infants are among the vulnerable groups that can be significantly affected by the poor air quality in which they live [12]. The attention to the adverse health outcome due to intrauterine PM exposure was illustrated after the 2008 Beijing Olympic Games, when the air quality was improved during that short period which allowed the comparison of the birth outcome between those with and without in utero exposure to heavy air pollution [13].

PMs with a diameter of 2.5 μm or less (PM_2.5_) are of particularly high risk to human health, including that of the growing foetus [14,15,16,17], even more than the gas component in the polluted air [16]. As such, countries with relatively clean air, e.g., Australia, are still at risk of PM derived from traffic-related air pollution [18]. Those living within 50 to 500 m of main roads are at higher risk of chronic low-level PM exposure and the associated adverse health effects [19,20,21,22,23]. For example, it has been found that living less than 200 m from a major road, meaning exposure to traffic-related air pollution, causes an increased risk of developing asthma and low lung function in children [20,24,25,26]. The small size gives such PMs the advantage of accessing the bloodstream in the alveoli and passing blood organ barriers, including the blood–placental barrier [9]. As such, PM_2.5_ can potentially circulate in foetal blood, although the foetal level compared with the maternal level is currently unclear.

The toxicity of PM is due to the complex composition, depending on the source [18]. The common substances carried by PM found in urban and industrial areas include sulphates, carbon, polycyclic aromatic hydrocarbons, biological compounds and metals [11,16,27]. Even in countries and areas with relatively good air quality, extreme weather conditions due to the change in the climate can significantly increase PM mass concentration within a short period of time, such as sand storms and bush fires [28,29]. A recent *Nature* paper has suggested that the oxidative potential of PM may be the driver of its adverse health effects [30]. In fact, PM contains high levels of free radicals and oxidants, such as reactive oxygen species (ROS) (e.g., oxygen and hydroxyl radicals and other reactive forms of O_2_ such as superoxide anion and hydrogen peroxide) [30,31]. Several PM components also generate ROS, including transition metals, polycyclic aromatic hydrocarbons and volatile organic compounds [32,33]. PM sourced from non-exhaust traffic emission contain many transition metals (i.e., manganese, vanadium, copper and iron) that have redox properties with the potential to induce intracellular ROS production, which then activates inflammatory cells to produce more ROS [34,35]. This has been associated with higher oxidative stress and toxicity compared to other sources [34,36]. Oxidative stress may be involved in all PM-induced disorders in multiple organ systems, including the lung, cardiovascular system and liver, which activate the endogenous redox system [37,38,39,40].

While the general public is generally conscious about outdoor pollutions, indoor air pollution is another frequent location of PM exposure that can affect the residents’ health. Indoor PMs can come from outdoor with similar chemical composition and size as environmental PMs [41]. Household generated PM can also be due to daily activities, such as cooking, biomass burning and cleaning [42,43,44]. Bisphenol A (BPA), commonly used in plastic products, has been found in 95% of indoor dust samples [45,46]. Although its inhalation is less than ingestion, BPA may interfere with lipid metabolism and inflammatory responses to increase the risk of atherosclerosis [46,47].

While it is well accepted that high ambient PM levels correlate with the mortality rate, it is increasingly recognised that long-term exposure to even low level of PM (quite often considered as “safe level”) increases the risk of disorders in vital organ systems, including the heart, the lung and the brain [48,49]. Although not widely studied, PMs are now considered an in utero environmental toxin [9,50] and therefore of interest to this perspective paper. Here, we summarised the currently available evidence from a limited number of publications to raise the awareness of the needs for more comprehensive research into this currently understudied yet important health topic.

## 2. Disrupted Foetal Development

As an intrauterine toxin and a strong oxidant, maternal exposure to PM during pregnancy is associated with birth complications and long-term health consequences in the offspring, including abnormal organogenesis, premature and preterm birth, small for gestational age, impairment in newborn lung function and immune function, and increased risk of brain developmental disorders and cognitive disorders after birth [9,50,51,52,53]. However, this topic is still understudied, considering that there is no evidence of a safe exposure threshold of any of the air pollutants [14].

The ability of PM_2.5_ to cross the blood–placental barrier suggests that PM_2.5_ can circulate in foetal blood [9]. Therefore, it can be naturally postulated that PM may directly induce oxidative stress and inflammatory responses in the growing foetus and affect foetal development [9,50]. This theory has been supported by studies on umbilical blood in newborns with prenatal PM exposure, in which reduced endogenous antioxidant Superoxide Dismutase 2 and DNA oxidative stress damage are discovered consistently in mother–baby pairs [54,55]. In vitro studies using embryonic cells or trophoblast cells have discovered dose-dependent toxicities of PMs on cell cycle and viability [50,56,57]. PM exposure affects several pathways, including heightened oxidative stress, inflammatory response and endoplasmic reticulum stress, resulting in ROS-JNK/ERK-apoptosis and G0/G1 arrest pathways [50,56,57]. These studies have shed light on what can happen to the growing foetus if the mother lives in polluted air during pregnancy. The cellular powerhouse mitochondria are sensitive to oxidative stress induced damage; however, mitochondrial function and integrity are not affected by PM exposure in an in vitro study [57]. Interestingly, changes in mitochondrial DNA copy number and methylation have been found in the cord blood of babies born to mothers exposed to PM during pregnancy [50]. This may be inherited from mothers, instead of caused by in utero PM exposure. In addition, in utero exposure to fine ambient PM correlates with heightened placental oxidative stress and inflammatory responses with decreased placental mass and gene expression responsible for placental angiogenesis [50,53]. This may impair nutrient delivery to the foetus, leading to intrauterine underdevelopment [58]. As the developing foetus is highly vulnerable to in utero environmental changes, in addition to low birth weight, intrauterine PM exposure can also result in miscarriage and preterm birth [58].

In line with Barker’s hypothesis, low birth weight can lead to an adaptive catchup growth after birth, which increases the risk of obesity. It is not surprising to observe fast weight gain in mice with pre-conceptional exposure to high levels of PM. Xu and colleagues demonstrated that females born to animals exposed to PM_2.5_ only during preconception seem to be protected, where only males in the 1st generation (F1) experience intrauterine development and catchup growth after birth [59]. The same study suggested that this transgenerational transmission may be driven by the effect of PM on mitochondrial DNA in eggs, as exposure to PM_2.5_ only during gestation did not have the same effect as the pre-conceptional exposure; while only the daughters in the 1st generation pass the adverse effect to the 2nd generation [59].

However, the situation seems more complex in humans, whose mothers are normally exposed to PM during both pre-conceptional and gestational periods. In humans, only girls show this predicted trend, whereas boys with intrauterine exposure to a higher level of PM remain underweight in childhood [60]. This may suggest that there is an additive effect between pre-conceptional and gestational exposures or even postnatal exposure, as babies normally live in the same environment as mothers. Whether the effect is consistent until adulthood is unclear for now. The discrepancy between few animals and human data available to date may have been related to unphysiologically high doses of PM used in animal studies or the effects of daily activities and weather changes on the level variations of PM exposure in human.

## 3. Risk of Future Respiratory and Metabolic Disorders

Maternal PM_2.5_ exposure has been found to cause foetal inflammation and oxidative stress, which influence organ development, and therefore increase the offspring’s susceptibility to non-communicable diseases in adulthood, as foetal development is a critical window that influences adult disease susceptibility [9,58]. In utero PM_2.5_ exposure has been shown to cause mitochondrial damage due to the mother inhaling oxidants leading to increased oxidative stress in the intrauterine environment, which then can cause dysregulation of the foetal immune system and interruption to the genetic duplication process causing adverse birth and foetal health outcomes [9,61]. Thus, maternal PM_2.5_ exposure in humans has been linked to increased risks of childhood asthma in the offspring [9].

The associations between direct PM exposure and the development of insulin resistance, abnormal cholesterol/triglyceride levels and obesity have been reported [62,63]. To date, there have been very few studies investigating the impact of intrauterine PM exposure on the risk of future metabolic disorders. The very first study was conducted on hamsters in 1982 and showed that PM_2.5_ was able to cross the blood–placental barrier, therefore reaching the foetus in utero. It also showed that maternal PM_2.5_ caused a decrease in mitotic activity in the foetal liver [64]. The liver is a key metabolic organ and has several roles, including acting as a hub, connecting metabolically various tissues and thus governing and maintaining body energy metabolism and metabolism homeostasis [65,66]. As this was a study on foetal hepatic development conducted nearly 40 years ago, this information is not up to date and is limited.

More recently, another study in 2019 discovered that prenatal and postnatal (4 weeks) PM_2.5_ exposure increased lipogenesis and worsened fatty acid oxidation differentially in mice consuming chow and high-fat diet [67]. Moreover, another study using continued PM exposure throughout development in mice showed transcriptomic changes in the liver in adulthood [68]. A pre-reviewed paper in BioRxiv showed maternal exposure to PM_2.5_ increased DNA methylation in pancreatic islets associated with the reduced blood insulin level and hyperglycaemia, which is an effect lasting for two generations [69]. Although no other work has supported the abovementioned discoveries, the above evidence suggests that foetal programming of metabolic disorders can be induced by intrauterine PM exposure. This needs to be confirmed by future studies.

## 4. Influence on Neurocognitive Function

Exposure to environmental toxins in utero can interrupt brain development [70]. PM may interfere with the formation of brain structures and cause failure in cell proliferation and the inability to modulate neurotransmission due to dysregulated pruning (loss of synapses) [71,72,73]. School-aged children who were exposed to high PM levels during their foetal life presented a thinner cortex in both hemispheres of the brain, particularly the precuneus region in the right hemisphere, which correlates with impaired inhibitory control [51]. In rats, maternal PM exposure led to decreased levels of IL-18 and vascular endothelial growth factor (VEGF) that are correlated with increased anxiety later [74]. These findings emphasise the links between intrauterine PM exposure and neurocognitive impairment [74]. Studies also suggest prenatal exposure to traffic PM_2.5_ may cause social behavioural changes by promoting pro-apoptotic pathways in the cerebral cortex during brain development [75].

PM may directly target the immune system by triggering glial cells, e.g., microglia, oligodendrocytes and astrocytes [71,72,73]. Microglia are resident innate immune cells within the central nervous system that respond to stimuli (cell stress, tissue damage, pathogens, etc.) and serve an active role in inflammation [76,77]. Although without direct evidence, intrauterine PM exposure may induce a similar inflammatory response in the brain regions as that of direct PM exposure (e.g., frontal cortex, substantia nigra, vagus nerve and the olfactory bulb) [78,79]. Elevated inflammation in the brain is also associated with blood–brain barrier leakage, leading to increased iron deposition in the brain and microbleeds [80]. Microbleeds are often associated with an impaired cognitive function, which may be responsible for the heightened risk of dementia due to direct PM exposure [81,82]. However, whether intrauterine PM exposure can lead to early-onset neurodegeneration and increased risk of dementia and other neurological conditions is unclear, which can be the focus of future epidemiological studies.

## 5. Disturbance on Body Fluid Homeostasis

Chronic exposure to PM has been associated with reduced kidney function [83,84,85,86,87]. The adverse impact of PMs on sodium excretion, natriuretic and diuresis further increases the risk of hypertension in such individuals [88,89]. An animal study suggests that in utero PM exposure can reduce renal dopamine D1 receptor function, which further leads to increased blood pressure driven by increased ROS production [89]. However, there is no literature to date to suggest the impact of intrauterine PM exposure on early kidney development and later susceptibility to renal dysfunction and chronic kidney disease (CKD) in adulthood.

A human study suggests that individuals who are exposed to PM from polluted air during foetal development have low birth weight and individuals with foetal underdevelopment have a 70% increased risk for CKD [90]. This is most likely driven by epigenetic modifications, which change DNA-encoded gene expression without affecting the original nucleotide sequence [91]. DNA methylation is the most widely studied epigenetic modification, with numerous studies linking its role to the development of CKD due to in utero environmental influences such as maternal cigarette smoking [2,92]. However, whether intrauterine PM exposure can program the risk of CKD via epigenetic modification is yet to be determined. Similar to PM, chemicals in cigarette smoke are also intrauterine toxins [2]. Intrauterine exposure can induce oxidative stress and inflammatory responses, which is linked to mitochondrial DNA damage, impaired mitochondrial function and structure and increased global DNA methylation in adult kidneys [2]. As a result, hallmarks of CKD have been found in these mice, including increased renal fibrosis and proteinuria [2,8,93]. Whether in utero PM exposure also induces CKD in adulthood through similar mechanisms is unclear. This requires future studies to close the knowledge gap.

## 6. A Temporary But Plausible Solution

Epidemiological studies have suggested that reducing PM exposure or the level of air pollution can reduce the risk of a variety of health problems [94]. Premature deaths could also be reduced by lowering air pollution to the WHO standard [14]. A study in India shows that life expectancy would increase by 1.7 years if PM levels are below that associated with adverse health outcomes [95]. This reduction in PM concentration is achievable through local and national governments establishing multisectoral policies in sectors such as transport, energy, agriculture, waste management and urban planning [11,96,97]. However, this goal is not easy to achieve. This largely depends on the willingness of the individual government to change their carbon emission policy and the influence of the surrounding countries. However, the health risks need to be addressed now.

The responses to prenatal PM exposure are comparable to cigarette smoke exposure, another common intrauterine oxidant/toxin. Both lead to oxidative stress and intrauterine underdevelopment [58,98]. Some of the long-term outcomes are also similar between these two stimuli [5,99,100], suggesting common pathological mechanisms and perhaps shared preventative solutions. We have shown that maternal supplements with either global antioxidant (e.g., L-carnitine) or mitochondria-targeted antioxidant (e.g., MitoQ) can ameliorate the detrimental impact of intrauterine cigarette smoke exposure caused foetal underdevelopment and risks of non-communicable disorders in multiple organ systems [92,101,102,103,104,105]. These benefits include the endocrine system that can lead to diabetes, the liver that can lead to dyslipidemia and liver steatosis, the brain that can lead to motor and cognitive dysfunction, the kidney that can lead to CKD and the lung that can lead to fibrosis and asthma [92,101,102,103,104,105]. Such effects are perhaps not restricted to suppressing oxidative stress in the growing foetal, as maternal vitamin C supplement during pregnancy has been shown to interrupt unwanted epigenetic modifications that lead to adverse health outcomes after birth due to intrauterine toxin exposure [106,107]. It is not clear whether administration of global or mitochondrial specific antioxidants during the gestation or early postnatal period can ameliorate adverse effects due to maternal PM exposure. Future studies addressing these issues warrant further investigations.

## 7. Perspective

Foetal development determines future health outcomes, in accordance with Barker’s hypothesis [6,7,9,19,20,21,22,23]. Therefore, any impact that in utero PM_2.5_ exposure has on the foetus may be carried into adulthood, despite the currently limited number of studies on the effect of in utero exposure to PM_2.5_ on the foetus in this regard. In addition, as the general public is not aware of the danger of low PM levels in places where air quality is considered good (e.g., Australia), they will not actively avoid it. Therefore, more epidemiological studies are needed to raise the awareness of both the general public and policy-makers for urban planning. Furthermore, although research on the adverse impact of in utero exposure to tobacco cigarette smoke has suggested the second and third trimester as a critical window to cause foetal underdevelopment [108], which trimester is more important for in utero PM exposure is still unclear. Investigating this research question can be challenging in humans, as moving house or changing working environment during pregnancy is not a common choice among most pregnant women. Perhaps only animal experiments can help identify the critical window during foetal development and use pharmacological approaches to identify the involvement of oxidative stress and inflammation in the toxicity due to in utero PM exposure.

**Figure 1:**
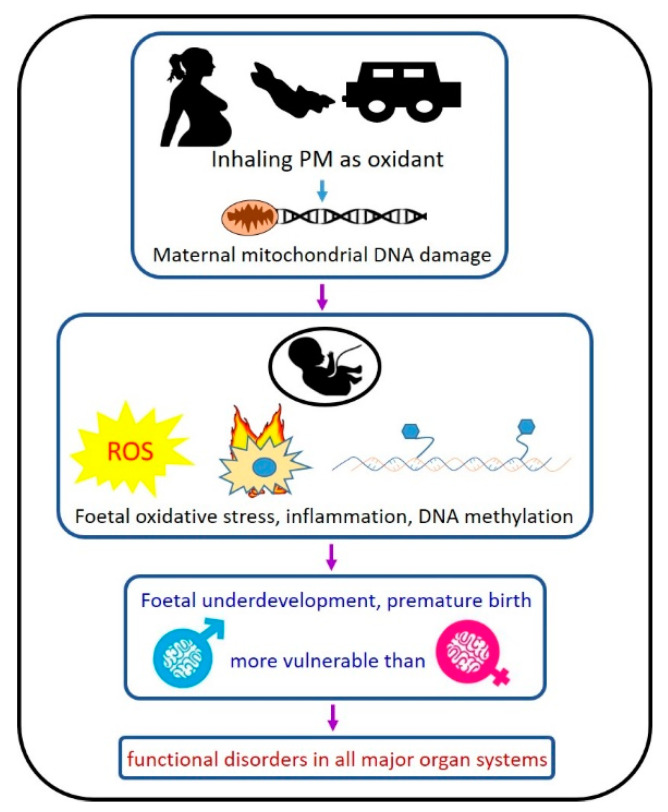
Proposed working mechanisms of how *in-utero* PM exposure leads to the future development of organ disorders.

In addition, in humans, newborns are more likely to live in the same polluted environment as their mothers, and thus postnatal development can be directly influenced by PM inhaled by their fragile lungs. Therefore, it is often difficult to separate the effects between in utero exposure and direct early-life inhalation. There have been some understandings of the respiratory and neurological effects of maternal PM exposure, whereas the impacts on the liver, kidney and cardiovascular are understudied. We have summarised the potential mechanisms in Figure 1 based on the published evidence. More studies are needed to examine how intrauterine PM exposure can interrupt normal organ development by adopting more physiologically relevant doses of PM. In addition, there is no safe limit for PM exposure. Future studies should also focus on the scenario of chronic low-level PM exposure in those with direct or in utero exposure.

Earlier investigations have established sexual bias in disease pathophysiology, with females less likely to develop certain diseases than males [109]. The conventional explanation is the anti-inflammatory effects of estrogen [110]. However, this is not always applicable to the sexual differences in foetal and early developmental disorders before puberty. Nevertheless, the sexual difference in the impact of intrauterine PM exposure has not been well studied, which may hold the key to develop a proper preventative strategy. 

## 8. Conclusions

Limiting pollution to reduce foetal and life exposure to PM is clearly the goal to achieve optimal health outcome. Maternal and early intervention to prevent chronic disease holds promise as a short-term solution; however, the effect of PM exposure during gestation on foetal health outcomes should be studied systematically. Additional studies are required to confirm whether oxidative stress is indeed the main mediator for disease development due to in utero PM exposure and identify the optimal foetal window for interventions and preventative measures.
